# Sequence-Dependent Modification of Bamboo Shoot Dietary Fiber Through Enzymatic Hydrolysis and *Lactobacillus plantarum* Fermentation

**DOI:** 10.3390/foods15122101

**Published:** 2026-06-11

**Authors:** Jingjing Du, Qian Zhu, Jiagang Guo, Jiayu Gu, Yuhan Wu, Jianlong Guo, Jian Jiang, Song Yang

**Affiliations:** 1 Institute of Agro-Products Processing, Anhui Academy of Agricultural Sciences, Hefei 230041, China; dujjmm@126.com (J.D.);; 2 Anhui Engineering Laboratory for Functional Microorganisms and Fermented Foods, Hefei 230041, China; 3 College of Food Science and Engineering, Anhui Science and Technology University, Chuzhou 239000, China

**Keywords:** bamboo shoot dietary fiber, sequential treatment, lactic acid bacteria fermentation, enzymatic hydrolysis, functional properties, in vitro digestion

## Abstract

Bamboo shoot dietary fiber (BSDF) is dominated by an insoluble fraction, which severely restricts its physicochemical performance and food application. In this study, the soluble dietary fiber (SDF) content of bamboo shoots was enhanced using three enzyme fermentation sequences: enzymatic hydrolysis followed by fermentation (EH), fermentation followed by enzymatic hydrolysis (F-EH), and integrated enzymatic hydrolysis and fermentation (IEHF). EH-F treatment resulted in the highest SDF content (17.27%). Variations in pH, biomass, enzyme activity, and short-chain fatty acids were assessed to understand the differences in the modification efficiency among the treatment sequences. Fourier transform infrared (FTIR) spectroscopy indicated that sequential enzymatic and fermentation treatments altered the chemical structure of bamboo shoot powder, consistent with the conversion of insoluble to soluble fractions. SDF from the EH-F treatment exhibited superior water-holding capacity (6.71 g/g), oil affinity (6.42 g/g), and DPPH radical scavenging rate (65.69% at 1.2 mg/mL). Moreover, SDF from the EH-F treatment achieved an 87.82% carbohydrate residue retention rate during simulated gastrointestinal digestion. These enhanced properties were associated with improved hydration properties resulting from the sequential tailoring process. This preliminary study explored the effects of different enzyme fermentation sequences on BSDF modification, providing a reference for the utilization of BSDF.

## 1. Introduction

Bamboo shoots are an abundant lignocellulosic biomass rich in dietary fiber, polysaccharides, and bioactive compounds. They have recently attracted increasing attention as a potential functional food ingredient [1,2]. Bamboo shoot dietary fiber (BSDF) exhibits various physiological activities, including hypoglycemic effects [3], modulation of gut microbiota [4], and antioxidant capacity [5]. However, native BSDF mainly consists of insoluble dietary fiber (IDF), which has a compact and highly ordered lignocellulosic structure [2,6,7,8], resulting in poor hydration properties, low fermentability, and limited functionality in food systems. Therefore, effective modification strategies are required to improve the soluble dietary fiber (SDF) content and functional properties of BSDF [9,10] and improve the physicochemical properties of plant dietary fibers. BSDF is mainly composed of cellulose and hemicellulose; therefore, cellulase and xylanase are considered effective enzymes for disrupting the fiber matrix and promoting the conversion of IDF into SDF [11,12]. However, enzymatic hydrolysis alone often results in incomplete structural transformations and limited functional improvements. Lactic acid bacteria (LAB) fermentation is another promising approach for dietary fiber modification. In particular, *Lactobacillus plantarum* is widely used in food fermentation because of its strong acid-producing ability, high adaptability to plant substrates, and safe (GRAS) status. During fermentation, *L. plantarum* produces organic acids and extracellular enzymes that can partially degrade polysaccharide structures and modify fiber surface characteristics, thereby improving hydration behavior and antioxidant activity [13,14,15].

Recent studies have demonstrated that enzymatic hydrolysis and microbial fermentation can exert synergistic effects on dietary fiber. Enzymatic pretreatment effectively loosens the compact fiber structure and enhances substrate accessibility, whereas subsequent microbial fermentation further remodels the polysaccharide architecture and produces diverse functional metabolites. Combined enzyme fermentation treatments can significantly improve the hydration performance, fermentability, and biological activity of plant dietary fibers. However, most studies have focused primarily on single modification strategies or simultaneous enzyme fermentation treatments [16,17]. The structural transformation differences, SDF formation mechanisms, and functional property variations of BSDF in response to different treatment sequences remain unclear, and the sequence-dependent synergistic mechanism underlying composite modification has not been systematically elucidated. Importantly, enzymatic hydrolysis and microbial fermentation have distinct optimal conditions in terms of pH, temperature, and substrate adaptability, implying that the synergistic treatment sequence serves as a decisive factor affecting fiber modification efficiency, IDF-to-SDF conversion, and functional metabolite accumulation.

In this study, three enzyme-fermentation combined sequences, namely enzymatic hydrolysis followed by fermentation (EH-F), fermentation followed by enzymatic hydrolysis (F-EH), and integrated enzymatic hydrolysis and fermentation (IEHF), were used to modify BSDF. This study investigated the intrinsic mechanisms underlying the changes in SDF content under different treatment sequences by monitoring dynamic changes in pH, biomass, enzyme activity, and short-chain fatty acids (SCFAs) during the treatment processes. Subsequently, the SDF fractions from each modified bamboo shoot group were isolated and subjected to physicochemical characterization, antioxidant activity analysis, and in vitro gastrointestinal digestion stability assessment. The aim of this study was to clarify the sequence-dependent synergistic effect of enzyme fermentation modification to provide a reference for the high-value development and industrial application of BSDF.

## 2. Materials and Methods

### 2.1. Materials

Fresh bamboo shoots (Moso bamboo) were purchased from Guangde Jingshi Agricultural Co., Ltd. (Xuancheng, China). The fresh bamboo shoots were washed, sliced, dried, and ground into powder that was passed through a sieve with 80 mesh. The resulting powder had a moisture content of 5.73%. Cellulase (EC 3.2.1.4, activity ≥10,000 U/g) and xylanase (EC 3.2.1.8, activity ≥20,000 U/g) were purchased from Shanghai Taixing Industrial Co., Ltd. (Shanghai, China). Both enzymes were of food-grade quality. Thermostable α-amylase from *Bacillus licheniformis* (EC 3.2.1.1, ≥3000 U/mg), protease from *Bacillus amyloliquefaciens* (EC 3.4.21.62, ≥16 U/g), and amyloglucosidase from *Aspergillus niger* (EC 3.2.1.3, ≥300 U/mL) were purchased from Sigma-Aldrich (St. Louis, MO, USA). The obtained enzymes were employed in enzyme treatment and for the determination of dietary fiber (DF) content. 2-(N-morpholino) ethanesulfonic acid (MES) and tris (hydroxymethyl) aminomethane (TRIS) buffers used for dietary fiber extraction were obtained from Sigma-Aldrich (St. Louis, MO, USA). The LAB strain *Lactobacillus plantarum* GS2, as the fermentation microorganism, was obtained from our laboratory stock culture collection. The strain was stored at −80 °C in de Man, Rogosa, and Sharpe (MRS) broth supplemented with 20% (*v*/*v*) glycerol and was activated by two successive subcultures in MRS broth at 37 °C for 24 h before use. Analytical-grade chemical reagents, including anhydrous ethanol, acetone, sodium hydroxide (NaOH), and hydrochloric acid (HCl), were supplied by Shanghai Aladdin Biochemical Technology Co., Ltd. (Shanghai, China).

### 2.2. Sample Preparation

**Figure 1:**
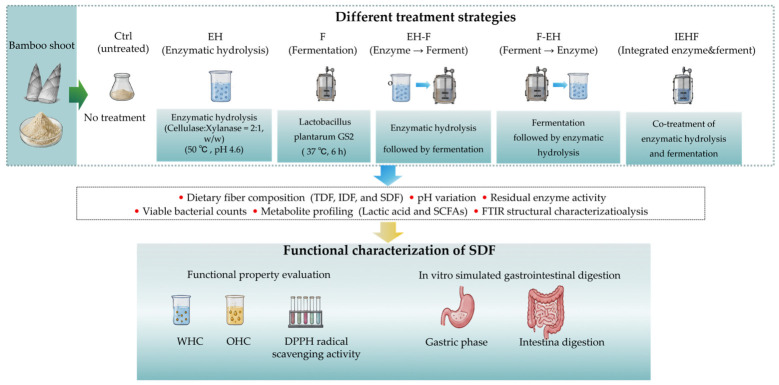
Schematic illustration of the different treatment strategies applied to bamboo shoot powder. TDF, total dietary fiber; IDF, insoluble dietary fiber; SDF, soluble dietary fiber; SCFAs, short chain fatty acids.

Six experimental groups were established: blank control (Ctrl), single enzymatic hydrolysis (EH), single fermentation (F), EH-F, F-EH, and IEHF groups. The experimental design and treatment sequence are shown in Figure 1.

#### 2.2.1. Blank Control Group (Ctrl)

The Ctrl group was prepared by mixing bamboo shoot powder with distilled water at a 1:10 (*w*/*v*) ratio, centrifuged at 8000× *g* for 10 min, and dried at 45 °C. Enzymatic hydrolysis or fermentation was not performed in this group. No enzymatic or microbial treatments were applied to this group.

#### 2.2.2. Enzymatic Hydrolysis Group (EH)

Enzymatic hydrolysis conditions were adapted from those reported in previous literature [7,8]. Briefly, 10.00 g of bamboo shoot powder was suspended in 100 mL of 0.1 mol/L citrate buffer (pH 4.6). Cellulose hydrolysis was performed using a commercial mixture containing cellulase (EC 3.2.1.4) and xylanase (EC 3.2.1.8) at a weight ratio of 2:1. This ratio was selected based on our previous optimization study [12]. The enzyme mixture was added to the suspension at a total dosage of 0.4% (*w*/*w*, based on the dry substrate). The cellulase and xylanase activities were 12,000 and 21,000 U/g, respectively, as provided by the manufacturer. The actual enzyme loadings were approximately 32.0 U/g (cellulase) and 28.0 U/g (xylanase), respectively. Hydrolysis was carried out at 50 °C for 2 h in a shaking incubator at 120 rpm (Guohua, Changzhou, China). The reaction was terminated by heating at 90 °C for 10 min to completely inactivate the enzymes, followed by centrifugation at 8000× *g* for 10 min. The resulting precipitate was collected and dried at 45 °C.

#### 2.2.3. Fermentation Group (F)

The bamboo shoot powder was mixed with water at a 1:10 (*w*/*v*) ratio and sterilized at 121 °C for 15 min to terminate enzymatic activity and ensure microbial safety. Then, the powder was cooled to 37 °C. *L. plantarum* GS2 was activated in MRS broth at 37 °C for 24 h. The activated culture (≥1 × 10^8^ CFU/mL) was inoculated at a dosage of 5% (*v*/*v*) into the suspension, resulting in a final inoculum level of approximately 5 × 10^6^ CFU/mL. Fermentation was carried out under static conditions (microaerophilic) at 37 °C for 6 h. The fermentation broth was centrifuged at 8000× *g* for 10 min, and the precipitate was dried at 45 °C.

#### 2.2.4. Enzyme-Bacteria Synergistic Treatment Groups

EH-F: The bamboo shoot powder was subjected to enzymatic hydrolysis as described in Section 2.2.2 for 2 h. Then the samples were sterilized at 121 °C for 15 min and cooled to 37 °C. The LAB inoculum was aseptically added at a concentration of 5% (*v*/*v*). Fermentation lasted 6 h at 37 °C. Then, the sample was centrifuged at 8000× *g* for 10 min and dried at 45 °C.

F-EH: The bamboo shoot powder was sterilized at 121 °C for 15 min and cooled to 37 °C. Then, the sample was fermented for 6 h according to the method described in Section 2.2.3, and the temperature was adjusted to 50 °C. A composite enzyme was added to the sample at the same dosage and ratio as described in Section 2.2.2. After performing enzymatic hydrolysis for 2 h, the reaction was terminated by heating at 90 °C for 10 min. Then, the sample was centrifuged at 8000× *g* for 10 min and dried at 45 °C.

IEHF: The bamboo shoot powder was mixed with water at a 1:10 (*w*/*v*) ratio and sterilized at 121 °C for 15 min. Then, the suspension was cooled to 37 °C. The samples were simultaneously co-inoculated with the composite enzyme (0.4%, *w*/*w*) and LAB inoculum (5%, *v*/*v*). The mixture was first incubated at 37 °C for 6 h. Then, the temperature was elevated to 50 °C to sustain enzymatic hydrolysis for 2 h. Although this temperature is not suitable for LAB proliferation, microbial metabolic contribution mainly occurred during the initial co-incubation stage. The enzymatic hydrolysis was terminated by heating at 90 °C for 10 min. Then, the sample was centrifuged at 8000× *g* for 10 min and dried at 45 °C.

### 2.3. Analysis of Total, Insoluble, and Soluble Dietary Fiber Contents

The contents of IDF and SDF were determined using the enzymatic–gravimetric method according to the official AOAC method 991.43 [18]. All dietary fiber contents were expressed as dry weights. Total dietary fiber (TDF) content was calculated as the sum of IDF and SDF.

### 2.4. Analysis of Physicochemical Indicators

Changes in pH during all treatments were measured using a PHS-3C digital pH meter (Leici, Shanghai, China). Cellulase and xylanase activities were determined by measuring reducing sugar content using the 3,5-dinitrosalicylic acid (DNS) method [19]. Enzymatic activity was measured at 50 °C in pH 4.6 buffer for 30 min, and the absorbance of the resultant mixture was read to evaluate the amount of reducing sugars released. Residual enzyme activity was calculated using the following formula:

(1) Residual activity (%) = (A_t_/A_0_) × 100,

where A_t_ and A_0_ represent the enzyme activity (U/mL) at a given time and initial enzyme activity (U/mL), respectively.

### 2.5. Metabolite Analysis

The concentrations of lactic acid and SCFAs, including acetic acid, propionic acid, and butyric acid, were determined using high-performance liquid chromatography (HPLC) using an Agilent 1260 HPLC system (Agilent Technologies, Santa Clara, CA, USA) equipped with a UV detector, according to a previously reported method with slight modifications [20]. Broth samples were collected at the end of fermentation and centrifuged at 8000× *g* for 10 min. The supernatants were filtered through a 0.22-μm membrane filter prior to HPLC analysis. Separation was performed on a C18 reverse-phase column (250 mm × 4.6 mm, 5 μm particle size) maintained at 30 °C. The mobile phase consisted of 20 mM phosphate buffer solution (pH 2.5) and acetonitrile under gradient elution conditions. The flow rate was set at 1.0 mL/min, and the injection volume was 10 μL. Absorbance was detected at a wavelength of 210 nm. Quantification was performed using external calibration curves prepared from the analytical standards of lactic, acetic, propionic, and butyric acids. All the calibration curves showed good linearity (R^2^ > 0.999). Metabolite concentrations were normalized to the dry weight of the initial bamboo shoot powder.

### 2.6. Microbiological Analysis

Viable bacteria were counted according to the Chinese National Standard GB 4789.35-202023 with slight modifications [21]. Aliquots of 1 mL were collected aseptically at fixed intervals, serially diluted 10-fold with sterile physiological saline, and plated on MRS agar. Colony-forming units (CFU) were counted after anaerobic incubation at 37 °C for 48 h. Bacterial numbers were reported in log_10_ CFU/mL.

### 2.7. Structural Characterization

The chemical functionalities of the samples were analyzed via Fourier transform infrared (FTIR) spectroscopy using a Thermo Nicolet iS50 spectrometer (Thermo Fisher Scientific, Waltham, MA, USA). The FTIR spectra were obtained in the wavenumber range from 4000 to 500 cm^−1^ and a spectral resolution of 4 cm^−1^, and each spectrum was accumulated from 32 scans [22,23].

### 2.8. Preparation of Soluble Dietary Fiber Samples

This study also investigated the functional properties and digestive stability of the solubilized fiber fractions. SDF was isolated from each modified bamboo shoot group. The treated SDF samples used for subsequent functional and digestive analyses were prepared as described in Section 2.3. Briefly, the modified bamboo shoot powders were subjected to sequential enzymatic digestion using thermostable amylase (EC 3.2.1.1, ≥3000 U/mg) at 100 °C for 30 min, protease (EC 3.4.21.62, ≥16 U/g) at 60 °C for 30 min (pH 7.5), and amyloglucosidase (EC 3.2.1.3, ≥300 U/mL) at 60 °C for 30 min (pH 4.5) to remove starch and protein. The soluble fraction was precipitated overnight using four volumes of 95% ethanol (*v*/*v*) at room temperature. The resulting precipitate was collected via centrifugation (8000× *g* for 10 min), washed with 78% aqueous ethanol, 95% ethanol, and acetone, and lyophilized to obtain purified SDF samples for further validation.

### 2.9. Functional Property Evaluation

Water-holding capacity (WHC) was determined using a previously reported method with slight modifications [24]. A total of 0.5 g of SDF was mixed with 20 mL distilled water and allowed to hydrate for 24 h at room temperature. The suspension was centrifuged at 5500× *g* for 15 min and the supernatant was discarded. WHC was calculated from the weight of the remaining hydrated residue according to the following equation:

(2) $$\mathrm{WHC}\;(g/g)=\frac{m_{2}-m_{1}}{m_{1}}$$

where *m*_1_ is the mass of the dry sample (g) and *m*_2_ is the mass of the water-containing residue (g).

Oil-holding capacity (OHC) was measured by blending SDF (0.5 g) with soybean oil (20 mL), maintaining it at 25°C for 24 h, and centrifuging it at 5500× *g* for 20 min [6]. The OHC was calculated as follows:

(3) $$\mathrm{OHC}\;(g/g)=\frac{W_{2}-W_{1}}{W_{1}}$$

where *W*_1_ is the weight of the dry sample (g), and *W*_2_ is the weight of the residue containing oil (g).

DPPH radical scavenging assay: The in vitro antioxidant activity was determined using a DPPH radical scavenging assay [25]. Briefly, 0.1 mL of SDF solution (1 mg/mL) was mixed with 0.9 mL of DPPH ethanol solution (0.1 mL) and incubated in the dark for 30 min. Then, absorbance was read at 517 nm. The DPPH radical scavenging rate was calculated based on the above absorption values.

### 2.10. In Vitro Simulated Gastrointestinal Digestion

In vitro gastrointestinal digestion was simulated according to the harmonized INFOGEST 2.0 static procedure [26] with some adaptations to mimic particular physiological conditions. The oral step was omitted, and the simulation started directly from the gastric phase. Samples were mixed with simulated gastric fluid (SGF) containing porcine pepsin, and the pH was adjusted to 3.0. Gastric digestion was performed at 37 °C for 2 h under continuous stirring. After the gastric phase, simulated intestinal fluid (SIF) containing porcine pancreatin and bile extract was added, and the pH was adjusted to 7.0 to initiate the intestinal digestion phase, which was carried out at 37 °C for 2 h. Digested samples were collected at the end of the gastric and intestinal phases. Enzymatic reactions were terminated by heating at 90 °C for 10 min, followed by centrifugation at 4000× *g* for 15 min to separate the soluble fraction from the undigested residue.

The resulting supernatant and residue were analyzed to determine digestibility endpoints. The reducing sugar content of the supernatant was measured using the DNS method [19] at 540 nm and compared to a glucose standard curve. The total carbohydrate content of the residue was determined using the phenol-sulfuric acid method [27], with the absorbance measured at 490 nm and the amount of residue calculated using a glucose standard curve. The residue ratio was calculated to determine the undigested portion of each SDF sample, and the original total carbohydrate mass for each group (Ctrl or EH-F) was considered as the reference point. The following equation was used for this calculation:

(4) Residue ratio (%) = (total carbohydrate mass in the intestinal residue/total carbohydrate mass in the initial sample) × 100

### 2.11. Statistical Analyses

All values are expressed as the mean ± standard deviation (*n* = 3). Statistical analysis was performed using one-way analysis of variance in IBM SPSS 25.0. A *p*-value of less than 0.05 was considered statistically significant.

## 3. Results

### 3.1. Changes in TDF, IDF, and SDF Contents After Different Treatments

The effects of different treatments on the TDF, IDF, and SDF contents of bamboo shoot powder are presented in Table 1. Compared to the control group, all the treated samples showed a significant increase in their SDF content. SDF content increased from 6.57% in the control group to 14.28% and 13.79% in the EH and F groups, respectively, indicating that both EH and F facilitated the conversion of insoluble fiber into soluble fractions. Moreover, the EH group exhibited a significantly higher SDF content than the F group, suggesting that EH was more effective in promoting fiber solubilization under the present conditions. Meanwhile, all treatments resulted in a slight decrease in TDF content compared to the control group. This reduction may be attributed to the degradation of high-molecular-weight polysaccharides into low-molecular-weight sugars, oligosaccharides, and other non-fiber metabolites during enzymatic hydrolysis and fermentation [10,28]. The EH-F group exhibited the highest SDF content (17.27%) and lowest IDF content (46.74%), indicating that sequential EH followed by fermentation most effectively promoted fiber solubilization. Compared to the EH-F treatment, the F-EH group showed a lower SDF content (15.08%), although it remained significantly higher than that of the single-treatment groups. These results suggest that treatment sequence plays an important role in determining the efficiency of dietary fiber conversion. The enhanced SDF formation observed in the EH-F group may be associated with the preliminary disruption of the compact fiber matrix during enzymatic hydrolysis, which likely improves substrate accessibility for subsequent microbial fermentation [11,29]. In contrast, the relatively lower efficiency of IEHF treatment may indicate that the staged co-treatment process did not provide sufficient structural disruption prior to microbial action.

### 3.2. Dynamic Changes in pH During Different Treatments

The dynamic changes in pH under different treatments are presented in Table 2. The pH of the Ctrl group remained relatively stable at approximately 6.5 throughout the treatment period, indicating minimal spontaneous acidification in the absence of enzymatic hydrolysis or fermentation. In the EH group, the pH was maintained at around 4.6 during the hydrolysis stage (0–2 h) due to the use of pH-controlled buffer, which provided a stable environment for enzymatic catalysis. Conversely, all the fermentation groups exhibited a progressive decrease in pH, reflecting the metabolic activity of *L. plantarum*, and the accumulation of organic acids during fermentation [13,15]. In the F group, the pH rapidly decreased from 6.50 to 5.28 within the first 2 h and further declined to 4.59 after 6 h, indicating active bacterial growth and acid production.

Different pH evolution patterns were observed in the sequential treatment groups. In the F-EH group, the pH decreased to 4.61 after 6 h of fermentation, and then remained relatively stable during the subsequent enzymatic hydrolysis stage. The acidic environment generated during fermentation was close to the optimal pH range for cellulase activity, which may have contributed to the maintenance of enzymatic hydrolysis efficiency during the subsequent treatment stages [12]. The EH-F group exhibited distinct two-stage pH profiles. During the initial enzymatic hydrolysis stage (0–2 h), the pH remained stable at approximately 4.6. After fermentation was initiated, the pH continuously decreased from 4.25 at 3 h to 3.81 at 8 h, showing the most pronounced acidification among all the treatment groups. This suggests that enzymatic pretreatment may enhance substrate accessibility for subsequent microbial fermentation. In contrast, the IEHF group showed the slowest decline in pH throughout the treatment period. The pH remained at 5.23 after 6 h and 5.12 after 8 h (Table 2), which was significantly higher than that of the other fermentation groups. The relatively limited acidification observed in the IEHF group may indicate less efficient microbial metabolism and substrate conversion during co-treatment.

### 3.3. Organic Acid Production Under Different Treatment Strategies

The concentrations of lactic acid and SCFAs produced under different treatment strategies are presented in Table 3. Among all the groups, the EH-F treatment generated the highest lactic acid content (42.56 mg/g), and its total SCFA content was higher than that of the F group. This result indicates that the EH-F strategy maintained active microbial metabolism during fermentation despite the relatively low initial pH conditions generated after enzymatic hydrolysis. The enhanced metabolite production observed in the EH-F group may be associated with structural modifications during the preliminary enzymatic hydrolysis stage. Enzymatic pretreatment may partially disrupt the compact fiber matrix, thereby improving the substrate accessibility for *L. plantarum* during the subsequent fermentation process [11,29]. Sustained bacterial growth (Table 4) further reflects the active metabolic state of the EH-F group.

In addition, the accumulation of lactic acid and other organic acids may contribute to the further modification of the fiber structures, which could be associated with the enhanced SDF formation shown in Table 1. In contrast, the IEHF group showed the lowest production of metabolites, including lactic acid (25.83 mg/g) and total SCFAs (7.93 mg/g). The IEHF group exhibited both the slowest pH decline (5.12 at 8 h) and the lowest total SCFA production, confirming that the limited acidification is a direct result of reduced microbial metabolic activity under the integrated treatment conditions. This result is consistent with its relatively weak acidification behavior shown in Table 2 and the lower viable bacterial counts shown in Table 4. The co-treatment conditions may not have been fully favorable for efficient microbial fermentation and enzymatic hydrolysis, thereby limiting substrate conversion efficiency.

The F-EH group exhibited intermediate metabolite levels, with lactic acid production (38.51 mg/g) lower than that of the EH-F group but higher than that of the IEHF group. Correspondingly, the SDF content was intermediate among the sequential treatment groups (Table 1). These results suggest that the treatment sequence influences the coordination between enzymatic hydrolysis and microbial fermentation. Compared to the F-EH treatment, the EH-F strategy provided more favorable conditions for subsequent microbial metabolism and fiber solubilization.

### 3.4. Residual Enzyme Activity and Lactic Acid Bacteria Growth

Table 4 presents the residual activities of cellulase and xylanase after the different treatment processes, along with the growth behavior of *L. plantarum*, as shown in Table 4. These results demonstrated that the treatment sequence significantly influenced both enzyme stability and bacterial proliferation. As shown in Table 4, the F-EH group exhibited the highest residual cellulase and xylanase activities after enzymatic hydrolysis among the groups, reaching 70.81% and 75.64%, respectively, which were significantly higher than those of the EH group. This may be associated with the acidic environment generated during the preliminary fermentation stage. As shown in Table 2, fermentation reduced the system pH to approximately 4.6, which is close to the optimal pH range for cellulase and xylanase activities. Such conditions may contribute to maintaining a higher enzymatic activity during the subsequent hydrolysis process. However, the IEHF group showed lower residual enzyme activity than the sequential treatment groups. At 6 h, cellulase and xylanase residual activities were 65.44% and 70.27%, respectively, which further decreased to 58.28% and 61.76%, respectively, after 8 h of treatment. The relatively low enzyme stability observed in the IEHF group may indicate less favorable conditions for sustained enzymatic activity during the integrated treatment process.

The growth behavior of *L. plantarum* further supported the differences among the treatment strategies. As shown in Table 5, the F-EH group exhibited rapid bacterial proliferation during the fermentation stage, reaching 9.05 × 10^9^ CFU/mL at 6 h, which was comparable to that of the F group. This result indicates that the fermentation-first strategy provided favorable conditions for bacterial growth prior to enzymatic hydrolysis. Compared to the F and F-EH groups, the IEHF group showed consistently lower viable bacterial counts throughout the treatment process, indicating that the integrated treatment conditions were less favorable for microbial proliferation. This reduced microbial activity is consistent with the weaker acidification behavior observed in Table 2. In the EH-F group, bacterial growth was relatively slow during the initial fermentation stage following enzymatic hydrolysis. However, the viable count gradually increased during fermentation and reached 9.85 × 10^8^ CFU/mL at 8 h, indicating that *L. plantarum* maintained metabolic activity after enzymatic pretreatment. These results further suggested that the treatment sequence affected the coordination between enzymatic hydrolysis and microbial fermentation, which may have contributed to differences in SDF formation among the treatments.

### 3.5. FTIR Characterization of Structural Alterations After Different Treatments

**Figure 2:**
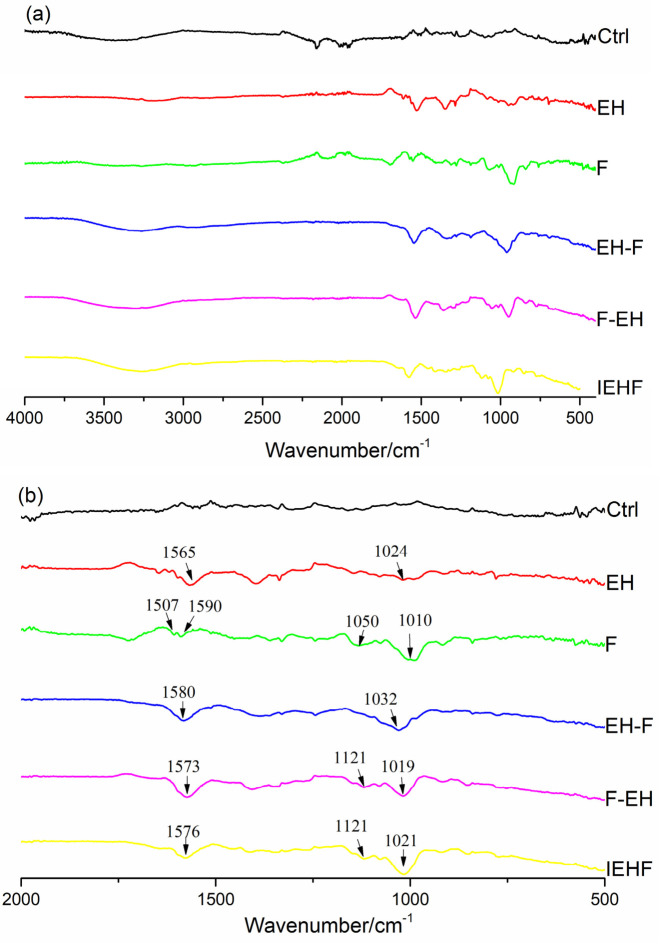
FTIR Spectra of bamboo shoot powder under different treatments: (**a**) full wavenumber range (4000–500 cm^−1^) and (**b**) expanded region (2000–500 cm^−1^).

FTIR spectroscopy was employed to characterize the structural variations in the bamboo shoot powder following different modification treatments (Figure 2a). The most pronounced spectral differences were observed in the wavenumber region of 500–2000 cm^−1^ (Figure 2b). Compared to the Ctrl group, all the treated samples exhibited noticeable spectral alterations after enzymatic hydrolysis, fermentation, or combined treatments, indicating that these processes modified the chemical environment of the fiber-associated components in the bamboo shoot powder.

The two representative spectral regions exhibited clear differences between the treatment groups. In the polysaccharide fingerprint region (1000–1200 cm^−1^), which is associated with C–O, C–C, and glycosidic bond vibrations of cellulose and hemicellulose, all the treated samples displayed enhanced absorption intensity and distinct peak shifts compared with the Ctrl group, suggesting that enzymatic hydrolysis and microbial fermentation altered the polysaccharide structure and glycosidic bond environment [23,29]. Specifically, the F, F-EH, and IEHF groups displayed two characteristic peaks (F: 1050 and 1010 cm^−1^; F-EH: 1019 cm^−1^ with a shoulder at 1120 cm^−1^; IEHF: 1021 cm^−1^ with a shoulder at 1120 cm^−1^). However, the EH-F group exhibited a single, broad peak centered at 1032 cm^−1^. This spectral difference indicates that sequential enzymatic pretreatment altered the polysaccharide structure in a distinct manner, which may be related to its higher SDF content.

In the lignocellulose-associated region (1500–1610 cm^−1^), different treatments also induced distinct spectral variations. The EH treatment caused a low-wavenumber shift to approximately 1565 cm^−1^, whereas the F treatment generated characteristic shoulder peaks at 1607 and 1590 cm^−1^. The combined treatment groups exhibited intermediate spectral features between those of the two treatment groups. These changes may reflect alterations in the lignocellulosic network and lignin–carbohydrate interactions [16], which could potentially increase the exposure of hydrophilic polysaccharide regions.

### 3.6. WHC and OHC of Modified SDF

**Figure 3:**
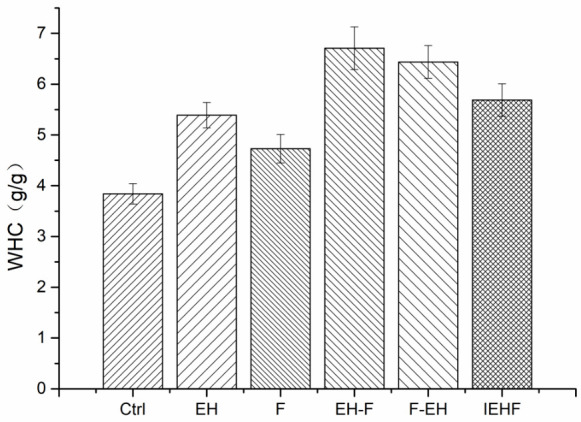
Comparison of the water-holding capacity (WHC) of SDF from bamboo shoots under different treatments.

**Figure 4:**
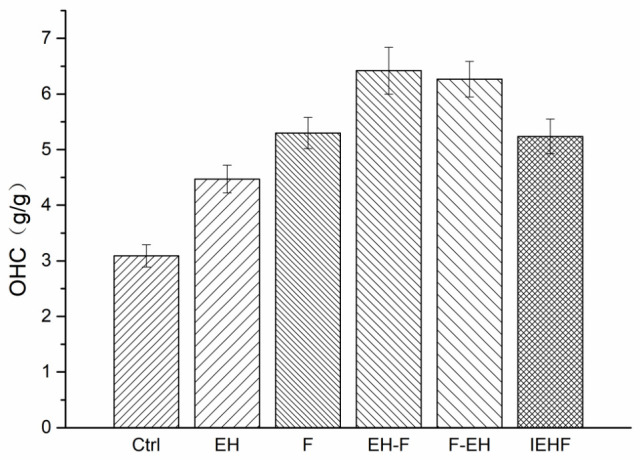
Comparison of the oil-holding capacity (OHC) of SDF from bamboo shoots under different treatments.

The WHC and OHC of the SDF fractions obtained from the different treatments are shown in Figure 3 and Figure 4. Compared with the Ctrl group, all the treatments improved both WHC and OHC, indicating that structural modification of dietary fiber enhanced the hydration and oil-binding properties of SDF. Among the single-treatment groups, the EH group exhibited a higher WHC than the F group, suggesting that enzymatic hydrolysis had a greater effect on modifying the fiber structure and hydrophilic properties. The EH-F group showed the highest WHC and OHC values, indicating that sequential enzymatic hydrolysis followed by fermentation is the most effective strategy for improving the functional properties of SDF. This enhancement may be associated with stepwise structural modifications that occurred during the sequential treatment. Enzymatic hydrolysis may initially disrupt the compact fiber matrix, whereas subsequent fermentation may further alter the polysaccharide network through microbial metabolism and acidification [15,28], thereby improving the WHC and OHC of the modified SDF. The reverse treatment sequence (F-EH) showed relatively low enhancement effects. This suggests that fermentation pretreatment may reduce the effectiveness of the subsequent enzymatic hydrolysis process. The IEHF group exhibited the lowest WHC and OHC among the combined treatment groups, which may be related to less favorable processing conditions during the integrated treatment process.

### 3.7. In Vitro Antioxidant Activity of Modified SDF

**Figure 5:**
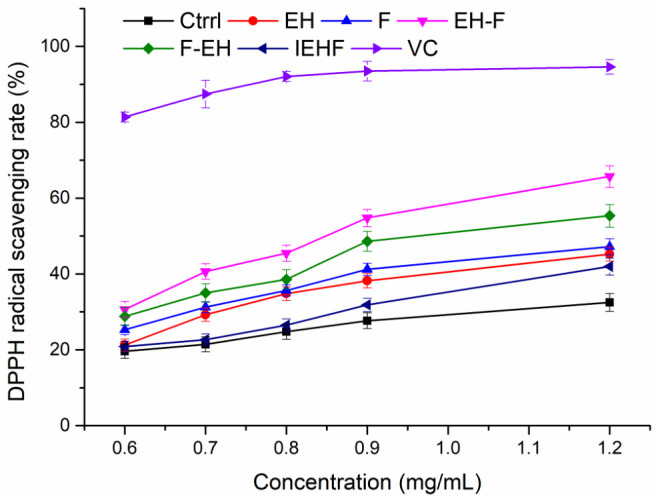
Comparison of the DPPH radical scavenging rates (concentration-dependent) of SDF under different treatments.

The in vitro antioxidant activities of the SDF obtained from the different treatment groups were evaluated using a DPPH radical scavenging assay (Figure 5). The DPPH radical scavenging activity of all samples increased with increasing SDF concentration, indicating a concentration-dependent antioxidant effect. The EH-F group consistently exhibited the highest radical-scavenging activity throughout the tested concentration range among all treatment groups. The scavenging rate of the EH-F group increased from 30.62% at 0.6 mg/mL to 65.69% at 1.2 mg/mL. At 1.2 mg/mL, the antioxidant activity followed the order EH-F > F-EH > F > EH > IEHF, indicating that sequential enzymatic hydrolysis followed by fermentation was the most effective treatment strategy for improving the antioxidant activity of SDF. Compared with the EH group, the F group exhibited higher DPPH radical scavenging activity at all tested concentrations, suggesting that microbial fermentation contributes more effectively to the enhancement of antioxidant capacity than enzymatic hydrolysis alone [15]. As shown in Table 2 and Table 5, the EH-F group exhibited the strongest acidification and the highest metabolite production, whereas the IEHF group showed relatively weaker acidification and lower metabolite levels. These differences may be associated with treatment-induced structural and physicochemical modifications of SDF during enzymatic hydrolysis and fermentation. The enhanced antioxidant activity observed in the EH-F group was consistent with its higher SDF content and improved functional properties, suggesting that treatment sequence significantly influenced the functional quality of the resulting SDF fractions.

### 3.8. In Vitro Gastrointestinal Stability of Modified SDF

EH-F treatment was identified as the optimal modification sequence for BSDF based on dietary fiber composition and physicochemical characteristics. SDF from the EH-F group was selected for in vitro simulated gastrointestinal digestion to evaluate its digestive stability, which was measured using carbohydrate residue retention. Native SDF (Ctrl) was used as a control. As shown in Table 6 and Table 7, the gastrointestinal behavior of the SDF was evaluated based on reducing sugar release and residual carbohydrate retention. During the gastric phase, both samples exhibited only slight changes in the reducing sugar content, which suggests that the polysaccharide matrix was relatively stable under acidic conditions. In contrast, pronounced differences were observed during the intestinal phase. The Ctrl group showed a marked increase in its reducing sugar concentration (89.36 mg/g), whereas the EH-F-treated SDF exhibited a significantly lower concentration (42.73 mg/g), indicating reduced enzymatic hydrolysis during intestinal digestion. Consistently, the residual carbohydrate ratio after digestion was higher in the EHF group (87.82%) than in the Ctrl group (70.36%), further confirming the improved structural resistance against enzymatic degradation. This observation agrees with previous reports showing that enzymatic fermentation combined with modification can enhance the gastrointestinal stability of dietary fibers by increasing their resistance to enzymatic hydrolysis [14]. These results indicated that EH-F modification reduced the extent of polysaccharide hydrolysis during the intestinal phase and promoted carbohydrate retention after digestion.

## 4. Conclusions

This study demonstrated that the sequence of enzymatic hydrolysis and fermentation played an important role in determining the structural and functional properties of BSDF. A comparative analysis of the EH-F, F-EH, and IEHF strategies showed that the order of process integration significantly influenced the conversion of insoluble dietary fiber into soluble fractions, resulting in an increase in SDF content from 6.57% to 17.27%. The EH-F group exhibited the most pronounced improvements in physicochemical properties, including enhanced WHC (6.71 g/g), OHC (6.42 g/g), and DPPH radical scavenging activity (65.69% at 1.2 mg/mL). In addition, in vitro digestion analysis suggested improved gastrointestinal stability, as evidenced by the reduced intestinal reducing sugar release (42.73 mg/g) and increased carbohydrate residue retention (87.82%). These findings suggest that the sequential combination of enzymatic hydrolysis and microbial fermentation contributes to the structural modification and functional enhancement of BSDF. Overall, the EH-F strategy provides an effective approach for improving the functional quality of BSDF and demonstrates its potential for application in functional food development. Nevertheless, further studies are required to optimize process efficiency and validate the applicability of the modified BSDF in real food systems.

## Figures and Tables

**Table 1:** TDF, IDF, and SDF contents of bamboo shoot powder under different treatments (%, dry basis).

Treatment Group	TDF	IDF	SDF
Ctrl	68.45 ± 1.20 ^a^	61.88 ± 1.05 ^a^	6.57 ± 0.25 ^f^
EH	67.23 ± 1.05 ^b^	52.95 ± 0.92 ^b^	14.28 ± 0.38 ^d^
F	65.87 ± 0.98 ^d^	52.08 ± 0.88 ^c^	13.79 ± 0.31 ^e^
EH-F	64.01 ± 1.08 ^f^	46.74 ± 0.82 ^f^	17.27 ± 0.52 ^a^
F-EH	65.55 ± 1.15 ^e^	50.47 ± 0.95 ^e^	15.08 ± 0.45 ^b^
IEHF	66.12 ± 1.11 ^c^	51.35 ± 0.90 ^d^	14.77 ± 0.35 ^c^

Note: Different lowercase superscript letters within the same column indicate significant differences (*p* < 0.05). Ctrl, Control group; EH, enzymatic hydrolysis; F, fermentation; EH-F, hydrolysis followed by fermentation; F-EH, fermentation followed by enzymatic hydrolysis; IEHF, integrated enzymatic hydrolysis and fermentation; TDF, total dietary fiber; IDF, insoluble dietary fiber; SDF, soluble dietary fiber.

**Table 2:** Dynamic changes in system pH during different treatment processes (Mean ± SD, n = 3).

Treatment Group	0 h	0.5 h	1 h	1.5 h	2 h	3 h	6 h	8 h
Ctrl	6.52 ± 0.05 ^a1^	6.51 ± 0.04 ^a1^	6.50 ± 0.06 ^a1^	6.50 ± 0.01 ^a1^	6.50 ± 0.04 ^a1^	6.50 ± 0.02 ^a1^	6.49 ± 0.04 ^a1^	
EH	4.61 ± 0.02 ^a3^	4.61 ± 0.02 ^a3^	4.60 ± 0.01 ^a3^	4.59 ± 0.02 ^a3^	4.59 ± 0.02 ^a3^	-	-	-
F	6.50 ± 0.04 ^a2^	6.01 ± 0.10 ^b2^	5.85 ± 0.08 ^c2^	5.57 ± 0.05 ^d2^	5.28 ± 0.03 ^e2^	5.15 ± 0.03 ^e2^	4.59 ± 0.02 ^f2^	-
EH-F	4.60 ± 0.04 ^a3^	4.60 ± 0.02 ^a3^	4.60 ± 0.03 ^a3^	4.60 ± 0.01 ^a3^	4.59 ± 0.02 ^a3^	4.25 ± 0.04 ^b3^	4.02 ± 0.03 ^c3^	3.81 ± 0.03 ^c3^
F-EH	6.51 ± 0.04 ^a2^	5.98 ± 0.09 ^b2^	5.82 ± 0.07 ^c2^	5.57 ± 0.02 ^d2^	5.29 ± 0.02 ^e2^	5.19 ± 0.02 ^e2^	4.61 ± 0.02 ^f2^	4.59 ± 0.01 ^f2^
IEHF	6.50 ± 0.05 ^a1^	6.05 ± 0.11 ^b2^	5.95 ± 0.08 ^c2^	5.85 ± 0.04 ^d2^	5.58 ± 0.03 ^e2^	5.35 ± 0.03 ^e2^	5.23 ± 0.03 ^f2^	5.12 ± 0.02 ^f2^

Note: EH, enzymatic hydrolysis only (0–2 h); F, fermentation only (0–6 h); EH-F, enzymatic hydrolysis (0–2 h) followed by fermentation (2–8 h); F-EH, fermentation (0–6 h) followed by enzymatic hydrolysis (6–8 h); IEHF, integrated enzymatic hydrolysis and fermentation (0–8 h). Values are expressed as the mean ± SD (n = 3). Different lowercase letters within the same row indicate significant differences over time (*p* < 0.05), whereas different superscript numbers within the same column indicate significant differences between treatments at the same time point (*p* < 0.05).

**Table 3:** Metabolite concentrations at the end of the fermentation stage in different treatment groups (mg/g, dry basis).

Treatment Group	Lactic Acid	Acetic Acid	Propionic Acid	Butyric Acid	Total SCFAs
F	35.22 ± 1.51 ^b^	8.11 ± 0.45 ^d^	1.22 ± 0.02 ^b^	0.86 ± 0.01 ^b^	10.19 ± 0.48 ^c^
F-EH (End of fermentation)	38.51 ± 1.20 ^b^	8.45 ± 0.31 ^b^	1.91 ± 0.07 ^a^	0.91 ± 0.02 ^b^	11.27 ± 0.65 ^b^
EH-F (End of fermentation)	42.56 ± 1.80 ^a^	9.30 ± 0.32 ^a^	2.07 ± 0.04 ^a^	0.97 ± 0.03 ^a^	12.34 ± 0.75 ^a^
IEHF (End of treatment)	25.83 ± 1.13 ^c^	6.59 ± 0.43 ^c^	1.02 ± 0.08 ^b^	0.32 ± 0.01 ^c^	7.93 ± 0.64 ^d^

Note: Different lowercase superscript letters within the same column indicate significant differences (*p* < 0.05).

**Table 4:** Residual activities of cellulase and xylanase at the end of different processing stages.

Treatment	Cellulase Residual Rate (%)	Xylanase Residual Rate (%)
EH (2 h)	68.52 ± 2.11 ^b^	72.34 ± 1.82 ^b^
EH-F (8 h)	N/A	N/A
F-EH (8 h)	70.81 ± 1.90 ^a^	75.64 ± 1.50 ^a^
IEHF (6 h)	65.44 ± 3.13 ^c^	70.27 ± 2.81 ^b^
IEHF (8 h)	58.28 ± 2.54 ^d^	61.76 ± 2.05 ^c^

Note: Different lowercase superscript letters within the same column indicate significant differences (*p* < 0.05). EH: Enzymatic Hydrolysis. N/A indicates that enzyme activity was not determined because of thermal inactivation prior to fermentation.

**Table 5:** Dynamics of viable lactic acid bacteria counts during different treatment processes (CFU/mL).

Treatment Time (h)	F	EH-F	F-EH	IEHF
0	(5.00 ± 0.20) × 10^6^	N/A	(5.00 ± 0.20) × 10^6^	(5.00 ± 0.20) × 10^6^
2	(3.50 ± 0.15) × 10^8^	(5.00 ± 0.20) × 10^6^	(3.80 ± 0.18) × 10^8^	(4.52 ± 0.22) × 10^8^
4	(8.91 ± 0.35) × 10^8^	(6.50 ± 0.25) × 10^7^	(8.94 ± 0.41) × 10^8^	(5.85 ± 0.40) × 10^8^
6	(9.02 ± 0.05) × 10^9^	(8.98 ± 0.37) × 10^8^	(9.05 ± 0.05) × 10^9^	(6.25 ± 0.06) × 10^9^
8	N/A	(9.85 ± 0.40) × 10^8^	N/A	N/A

Note: Values are expressed as mean ± SD (n = 3). N/A indicates not applicable due to transition to the enzymatic hydrolysis stage.

**Table 6:** Changes in reducing sugar content during in vitro digestion (mg/g).

Sample	Before Digestion (Initial)	After Gastric Phase	After Intestinal Phase
Ctrl	15.59 ± 0.08 ^a^	17.87 ± 1.34 ^a^	89.36 ± 4.20 ^b^
EH-F	13.86 ± 0.07 ^a^	14.54 ± 1.09 ^a^	42.73 ± 1.51 ^c^

Different superscript letters within the same row indicate significant differences (*p* < 0.05).

**Table 7:** Total carbohydrate residue ratio of SDF samples after the intestinal phase of in vitro digestion.

Sample	Total Carbohydrate Before Digestion (mg)	Total Carbohydrate in Intestinal Residue (mg)	Residue Ratio (%)
Ctrl	100.0 ± 2.5 ^a^	70.36 ± 3.1 ^b^	70.36%
EH-F	100.0 ± 2.5 ^a^	87.82 ± 2.8 ^a^	87.82%

Different superscript letters within the same column indicate significant differences (*p* < 0.05).

## Data Availability

The original contributions presented in this study are included in the article. Further inquiries can be directed to the corresponding authors.

## Abbreviations

The following abbreviations are used in this manuscript:

AOAC	Association of Official Analytical Chemists
BSDF	Bamboo shoot dietary fiber
CFU	Colony-forming unit
Ctrl	Control group
DNS	3,5-Dinitrosalicylic acid
DPPH	2,2-Diphenyl-1-picrylhydrazyl
EC	Enzyme Commission number
EH	Enzymatic hydrolysis
EH-F	Enzymatic hydrolysis followed by fermentation
F	Fermentation
F-EH	Fermentation followed by enzymatic hydrolysis
FTIR	Fourier-transform infrared spectroscopy
GI	Gastrointestinal digestion
GRAS	Generally Recognized as Safe
HPLC	High-performance liquid chromatography
IDF	Insoluble dietary fiber
IEHF	Integrated enzymatic hydrolysis and fermentation
LAB	Lactic acid bacteria
MES	2-(N-morpholino) ethanesulfonic acid
MRS	de Man–Rogosa–Sharpe
OHC	Oil-holding capacity
SCFAs	Short-chain fatty acids
SDF	Soluble dietary fiber
TDF	Total dietary fiber
TRIS	Tris(hydroxymethyl)aminomethane
WHC	Water-holding capacity

## References

[1] Li Q., Fang X., Chen Y., Han Y., Liu R., Wu W., Gao H. (2021). Retarding effect of dietary fibers from bamboo shoot (Phyllostachys edulis) in hyperlipidemic rats induced by a high-fat diet. Food Funct..

[2] Yang J., Wu L., Yang H., Pan Y. (2021). Using the Major Components (Cellulose, Hemicellulose, and Lignin) of Phyllostachys praecox Bamboo Shoot as Dietary Fiber. Front. Bioeng. Biotechnol..

[3] Zheng Y., Wang Q., Huang J., Fang D., Huang W., Luo X., Zou X., Zheng B., Cao H. (2019). Hypoglycemic effect of dietary fibers from bamboo shoot shell: An in vitro and in vivo study. Food Chem. Toxicol..

[4] Zhou X., Ma L., Li D., Li D., Chen F., Hu X. (2023). Bamboo shoot dietary fiber alleviates gut microbiota dysbiosis and modulates liver fatty acid metabolism in mice with high-fat diet-induced obesity. Front. Nutr..

[5] Chen X., Wang B., Li X., Yu L., Zhang Y. (2025). Enhancement of physicochemical properties and antioxidant activities of bamboo shoot dietary fibers via γ-irradiation. Food Chem. Adv..

[6] Cuidie T., Yinlai Y., Fusheng Z., Jianquan K., Jinguo W., Jiong Z. (2022). Insight into the physicochemical, structural, and in vitro hypoglycemic properties of bamboo shoot dietary fibre: Comparison of physical modification methods. Int. J. Food Sci. Technol..

[7] Song Y., Su W., Mu Y.C. (2018). Modification of bamboo shoot dietary fiber by extrusion-cellulase technology and its properties. Int. J. Food Prop..

[8] Tang C., Wu L., Zhang J., Kan J., Zheng J. (2022). Comparison of different extraction methods on the physicochemical, structural properties, and in vitro hypoglycemic activity of bamboo shoot dietary fibers. Food Chem..

[9] Mammolenti D., Lupi F.R., Baldino N., Gabriele D. (2025). Technological Advancements of Insoluble Dietary Fiber from Food By-Product Processing: A Review. Foods.

[10] Tang W., Lin X., Walayat N., Liu J., Zhao P. (2024). Dietary fiber modification: Structure, physicochemical properties, bioactivities, and application—A review. Crit. Rev. Food Sci. Nutr..

[11] Xing J., Zhang Z., Zhao Q., Li X., Zhao J. (2025). Influence of one-step enzymatic modification on the structure, physicochemical, and functional properties of dietary fiber from corn husk rich in (hemi)cellulose. Int. J. Biol. Macromol..

[12] Du J., Zhu Q., Guo J., Wu Y., Guo J., Yang S., Jiang J. (2025). Process Optimization and Functional Characterization of Bamboo Shoot Powder Modified by Composite Cellulase/Xylanase Hydrolysis. Food Res. Dev..

[13] Liao A.M., Zhang J., Yang Z.L., Huang J.H., Pan L., Hou Y.C., Li X.X., Zhao P.H., Dong Y.Q., Hu Z.Y. (2022). Structural, Physicochemical, and Functional Properties of Wheat Bran Insoluble Dietary Fiber Modified With Probiotic Fermentation. Front. Nutr..

[14] Wang X., Xue H., Li Y., Li P., Liu Z., Piao C. (2024). Characterization of physicochemical and functional properties of soluble dietary fiber from separate and co-fermented okara by lactic acid bacteria and Kluyveromyces marxianus C21. LWT.

[15] Li Y., Niu L., Guo Q., Shi L., Deng X., Liu X., Xiao C. (2022). Effects of fermentation with lactic bacteria on the structural characteristics and physicochemical and functional properties of soluble dietary fiber from prosomillet bran. LWT.

[16] Milić M.D., Bunić A.V., Mihajlović K.R., Ilić N.V., Davidović S.Z., Dimitrijević-Branković S.I. (2022). The development of a combined enzymatic and microbial fermentation as a viable technology for the spent coffee ground full utilization. Biomass Convers. Biorefin..

[17] Zeng H., Ding L., Hou M., Liu Z., Pan L., Hang S. (2025). Enhancing palm kernel cake nutritional quality through combined bacterial fermentation and enzymatic hydrolysis. J. Sci. Food Agric..

[18] Prosky L., Asp N.G., Schweizer T.F., Devries J.W., Furda I. (1988). Determination of insoluble, soluble, and total dietary fiber in foods and food products: Interlaboratory study. J. AOAC Int..

[19] Miller G.L. (1959). Use of Dinitrosalicylic Acid Reagent for Determination of Reducing Sugar. Anal. Chem..

[20] Serafim J.A., Silveira R.F., Vicente E.F. (2021). Fast determination of short-chain fatty acids and glucose simultaneously by ultraviolet/visible and refraction index detectors via high-performance liquid chromatography. Food Anal. Methods.

[21] (2023). National Food Safety Standard—Food Microbiological Examination—Lactobacillus Examination.

[22] Du J., Guo J., Guo Q., Zhu Q., Guo J., Gu J., Ren Y., Wu L., Yang S., Jiang J. (2025). Enhancement of Polyvinyl Alcohol-Based Films by Chemically Modified Lignocellulosic Nanofibers Derived from Bamboo Shoot Shells. Polymers.

[23] Kruer-Zerhusen N., Cantero-Tubilla B., Wilson D.B. (2018). Characterization of cellulose crystallinity after enzymatic treatment using Fourier transform infrared spectroscopy (FTIR). Cellulose.

[24] Gan J., Huang Z., Yu Q., Peng G., Chen Y., Xie J., Nie S., Xie M. (2020). Microwave assisted extraction with three modifications on structural and functional properties of soluble dietary fibers from grapefruit peel. Food Hydrocoll..

[25] Wolosiak R., Drużyńska B., Derewiaka D., Piecyk M., Majewska E., Ciecierska M., Worobiej E., Pakosz P. (2021). Verification of the Conditions for Determination of Antioxidant Activity by ABTS and DPPH Assays—A Practical Approach. Molecules.

[26] Brodkorb A., Egger L., Alminger M., Alvito P., Assunção R., Ballance S., Bohn T., Bourlieu-Lacanal C., Boutrou R., Carrière F. (2019). INFOGEST static in vitro simulation of gastrointestinal food digestion. Nat. Protoc..

[27] Dubois M., Gilles K.A., Hamilton J.K., Rebers P.A., Smith F. (1956). Colorimetric Method for Determination of Sugars and Related Substances. Anal. Chem..

[28] Wen Y., Niu M., Zhang B., Zhao S., Xiong S. (2017). Structural characteristics and functional properties of rice bran dietary fiber modified by enzymatic and enzyme-micronization treatments. LWT.

[29] Sarafidou M., Foys A., Godziek M., Kobyliukh A., Trzebicka B., Pispas S., Koutinas A., Tsouko E. (2025). Modification of Bacterial Nanocellulose Using Nonthermal Plasma-Assisted Enzymatic Hydrolysis. Biomacromolecules.

